# Low-dose mycophenolate mofetil improves survival in a murine model of *Staphylococcus aureus* sepsis by increasing bacterial clearance and phagocyte function

**DOI:** 10.3389/fimmu.2022.939213

**Published:** 2022-07-19

**Authors:** Fanny Alby-Laurent, Nadia Belaïdouni, Benoit Blanchet, Christophe Rousseau, Jean-François Llitjos, Sylvia Sanquer, Jean-Paul Mira, Frédéric Pène, Julie Toubiana, Jean-Daniel Chiche

**Affiliations:** ^1^ Cochin Institute, Department of Infection, Immunity and Inflammation, Inserm U1016, Paris Descartes Sorbonne Paris Cité University UMR-S1016, Centre National de la Recherche Scientifique (CNRS) UMR 8104, Paris, France; ^2^ Department of Pharmocology and Toxicology, Cochin Hospital, Assistance Publique des hôpitaux de Paris (APHP), Université de Paris, Paris, France; ^3^ Medical Intensive Care Unit, Cochin Hospital, APHP, Université de Paris, Paris, France; ^4^ Metabolic and Proteomic Biochemistry Department, Necker-Enfants malades Hospital, Université de Paris, Paris, France; ^5^ Department of General Pediatrics and Infectious Diseases, Necker-Enfants malades Hospital, APHP, Université de Paris, Paris, France; ^6^ Department of Intensive Care Medicine, Hospital and University of Lausanne, Lausanne, Switzerland

**Keywords:** mycophenolate mofetil, sepsis, staphylococcus aureus, NF-κB, toll-like receptor 4, innate immunity, macrophages, phagocytosis

## Abstract

Regulators of TLRs signaling pathways play an important role in the control of the pro-inflammatory response that contributes to sepsis-induced tissue injury. Mycophenolate mofetil, an immunosuppressive drug inhibiting lymphocyte proliferation, has been reported to be a regulator of TLRs signaling pathways. Whether MMF used at infra-immunosuppressive doses has an impact on survival and on innate immune response in sepsis is unknown.

C57BL/6J mice were infected intraperitoneally with 10^8^ CFU *Staphylococcus aureus*, and treated or not with low-dose of MMF (20mg/kg/day during 4 days). Survival rate and bacterial clearance were compared. Cytokine levels, quantitative and qualitative cellular responses were assessed. *S. aureus* – infected mice treated with MMF exhibited improved survival compared to non-treated ones (48% vs 10%, p<0.001). With the dose used for all experiments, MMF did not show any effect on lymphocyte proliferation. MMF treatment also improved local and systemic bacterial clearance, improved phagocytosis activity of peritoneal macrophages resulting in decreased inflammatory cytokines secretion. MMF-treated mice showed enhanced activation of NF-κB seemed with a suspected TLR4-dependent mechanism. These results suggest that infra-immunosuppressive doses of MMF improve host defense during *S. aureus* sepsis and protects infected mice from fatal outcome by regulating innate immune responses. The signaling pathways involved could be TLR4-dependent. This work brings new perspectives in pathogenesis and therapeutic approaches of severe infections.

## Introduction

Sepsis remains a leading cause of critical illness and mortality worldwide despite recent advances in supportive care. Recently redefined as life-threatening organ dysfunction due to a dysregulated host response to infection (1), sepsis involves profound alterations of the innate immune response. Recognition of the invading pathogen triggers an innate response that involves a cascade of cellular and molecular events in order to eradicate the responsible microorganism. At the early stage of infection, the pro-inflammatory response that aims to clear pathogen also has a “fitness cost” for the host as it can result in organ dysfunction, failure and subsequent death (2). A tight regulation of the innate immune response is necessary to tip the balance toward efficient phagocytosis and pathogen killing while avoiding or limiting organ failure (3). Immunomodulating agents such as monoclonal antibodies targeting pro-inflammatory cytokines have been tested in order to restore this balance and improve sepsis outcomes. They have failed to reduce mortality in patients with sepsis and septic shock (4, 5), possibly because a potent pro-inflammatory response is essential to efficient pathogen clearance. In support of this hypothesis, autopsy results have shown that most patients that died with sepsis had unresolved septic foci (5), suggesting that failure of the host’s immunity to eradicate invading pathogens is closely associated with mortality.

Approaches that could improve protective anti-infective immunity while limiting sepsis-induced tissue injury might increase survival in sepsis (6). By modulating innate immune responses, negative regulators of TLRs signaling pathways play an important role in the control of the pro-inflammatory response that contributes to sepsis-induced tissue injury (7). Mycophenolate mofetil (MMF), an immunosuppressive drug used to prevent graft rejection after transplantation, and its active substrate mycophenolic acid (MPA) have been reported to be negative regulators of TLRs signaling pathways (8, 9). The effects of MMF during sepsis have been tested only in two experimental studies. Huang et al. have recently reported that large doses of MMF given at the time of infection improve survival in a murine model of sepsis (10), whereas one study showed that long-term (7-days) treatment with infra-immunosuppressive doses of MMF before polymicrobial sepsis failed to reduce mortality (11). These conflicting results might be related to the targeted MMF concentrations and their impact on immune functions.

The immunosuppressive effects of MMF on adaptive immunity have been firmly established. MMF blocks T and B-cell proliferation by inhibiting inosine monophosphate deshydrogenase II (IMPDHII). These effects are dose-dependent and usually monitored by measuring IMPDHII activity or MPA residual concentrations (C_0_). Indeed, MPA C_0_ correlates with graft rejection and adverse events such as viral reactivations and infections or leucopenia. Target concentrations for adequate immune suppressions range between 1,5 - 2mg/L, as lower C_0_ is associated with acute graft rejection and C_0_ > 2mg/L increases the risk of leucopenia (12). Recent data suggest that MMF also has an impact on innate immune responses, as shown by its ability to blunt release of proinflammatory cytokines (12). Interestingly, MMF does not increase the incidence of bacterial infections in patients treated for prevention of graft rejection (12).

We hypothesized that MMF used at infra-immunosuppressive concentrations (C_0_ < 1,5mg/L) could modulate pro-inflammatory response during bacterial infections and prevent tissue injury without having a negative impact on bacterial clearance. To test whether low concentrations of MMF have an impact on innate immune responses and survival in sepsis, we investigated the effect of low-dose MMF in a model of gram-positive bacteremia through a peritoneal injection of *S. aureus* (13). We report that MMF treatment improves survival in septic animals and enhances clearance of *S. aureus* through its impact on innate immune responses.

## Materials and methods

### Mice and ethics statement

Female C57BL/6J mice aged 8–12 weeks were used in all experiments. Wild-type mice were purchased from Charles River Laboratories. Knock-out Tlr2(-/-) and Tlr4(-/-) mice were obtained from S. Akira (Osaka University, Osaka, Japan) and were backcrossed 8 times with C57BL/6J mice. Animals were maintained in the pathogen-free animal facility of the Cochin Institute. Experiments were conducted in compliance with European animal welfare regulation the (3R) and was approved by the Institutional Animal Care and Use Committee and by the French Agriculture and Forestry Ministry (APAFiS #12959).

### Bacterial strains & growth conditions

*S. aureus* strain Newman was grown in tryptic soy broth (TSB). Overnight cultures of *S. aureus* were diluted 1/100 into fresh TSB and grown for 2 h at 37°C. The cultures were sedimented, washed, and suspended in phosphate buffered saline (PBS) to obtain an inoculum of 5.10^8^ CFU/mL. Mycophenolic acid (MPA) (Sigma Aldrich, Germany) was used *in vitro* to detect any direct effect on bacterial growth. In order to obtain heat-killed *S. aureu*s (HKSA), culture of *S. aureus* was boiled at 95°C.

### Model of infection

Mice were infected *via* an intraperitoneal (i.p). injection of 10^8^ CFU *S. aureus*. Inocula were determined by CFU numeration following serial dilution, plating in tryptic soy agar, and growth at 37°C. This model results in bacteremia (bloodstream infection) in 100% of the animals (data not shown).

### MMF treatment protocol

Lyophilized MMF powder (Cellcept^®^, Roche) was reconstituted in NaCl 0.9% and diluted to a concentration of 2 mg/mL. The vehicle control solution for *in vivo* experiments consisted of NaCl 0.9%. Each mouse received an intraperitoneal (i.p.) injection of 20mg/kg (corresponding to 100μl/g) of MMF or vehicle control solution of the same volume every 24 h for 5 days. Treatment started one day before the i.p. injection of *S. aureus*.

### Isolation of peritoneal macrophages, splenic cells, and peripheral blood mononuclear cells

Mice were euthanized, and 20 mL of RPMI was injected in the peritoneal cavity using a 26g needle. The peritoneum was massaged and the fluid was then collected using a 26g needle attached to a 10 mL syringe. If visible blood contamination was detected, the contaminated sample was discarded. Cell suspensions were then centrifugated at 1600 rpm during 6 minutes and cells pellets were resuspended in RPMI. Cells were counted and put in a 24-well plate at the number of 600,000 cells/plate. After 2 hours of adhesion, supernatant was removed and macrophages (adherent cells) were then recovered. Splenic cells were isolated after spleen digestion at 37°C during 10 minutes with supplemented RPMI (RPMI 1640 + Glutamax, decomplemented SVF 10%, Pyruvate sodium 1%, Penicillin S 1%, non essential amino-acids 0,01% and β-mercaptopurine 0,2%) with DNAse (10 mg/mL 1%) and D-collagenase (4 mg/mL 25%), filtrated on a cellular tamis (40 μm) and centrifugation at 1600 rpm during 6 minutes. PBMCs were isolated from mice heparinized blood obtained by cardiac puncture under anesthesia (i.p. ketamine (100mg/kg) and xylazine (10mg/kg)). Whole blood was mixed with NaCl 0.9% and layered over Ficoll-Paque™ PLUS (GE Healthcare, England) (2mL blood over 2mL Ficoll) and centrifuged according to the manufacturer’s protocol (30 min at room temperature).

### Mycophenolic acid plasmatic residual concentration measurement

Plasmatic residual concentrations of MPA were measured every 24 hours. Blood was obtained from cardiac puncture of anesthetized mice (i.p. ketamine (100mg/kg) and xylazine (10mg/kg)), and enzyme-multiplied immunoassay technique (EMIT) based on inosine monophosphate deshydrogenase inhibition was used to measure plasmatic concentrations of mycophenolic acid, as previously described (14).

### Measurement of IMPDH activity

IMPDH isoform II (IMPDHII) is a key enzyme of the *de novo* guanosine synthesis in hematopoietic stem cells through the NAD-dependent conversion of inosine monophosphate (IMP) to xanthosine monophosphate (XMP) (15). We measured IMPDH activity in in PBMCs and in peritoneal macrophages by measuring the transformation of NAD in NADH + H+ and of IMP in XMP with Ultra High Pressure Liquid chromatography-mass spectrometry (16). We measured IMPDHII activity in mice treated with MMF and control mice, 4 days after treatment with MMF (or vehicle), one hour after the last injection.

### Measurement of NF-κB activity

THP1-Blue™ NF-κB cells (*In vivo*Gen, San Diego, CA) were grown in RPMI 1640 with 2 mM L-glutamine, 25 mM HEPES, 10% FBS, 100 μg/ml Normocin, and 1% penicillin/streptomycin and then plated at the number of 2 x 10^5^ cells per well in a 96 well plate in a medium with blasticidin and MPA at different concentrations. Cells were then stimulated with 1 x 10^5^ of heat-killed *S. aureus* (HKSA) or Lipopolysaccharide (LPS) and incubated at 37°C in 5% CO_2_ during 24 h. Cell culture supernatants were incubated with QUANTI-Blue medium (*In vivo*gen, rep-qb1), and alkaline phosphatase activity was measured (optical density 620 nm).

### Determination of bacterial load in peritoneal cavity and blood

Blood and peritoneal fluid were collected 6 and 24 hours after infection and were subjected to serial 10-fold dilutions. Bacteria were quantified in tryptic soy agar after 24h at 37°C and controlled to confirm positive catalase activity.

### Serum cytokines measurement

Heparinized blood from treated and non-treated mice was collected by cardiac puncture 0, 6 and 24 hours after *S. aureus* infection. Samples were then centrifuged at 4000rpm during 5 minutes in order to collect supernatant. Supernatants were stored at −80°C until evaluated for cytokine secretion by Meso Scale Discovery (Rockville, Maryland, USA) technology, using a mouse V-PLEX Plus Proinflammatory Panel 1 kit providing assay-specific components for the quantitative determination of INF-γ, IL-1β, IL-2, IL-4, IL-5, IL-6, KC, IL-10, IL-12p70 and TNF-α. Kits were run according to the manufacturer’s instructions. Samples were run in duplicate.

### Isolation of peritoneal macrophages and splenic cells

Peritoneal cells were collected after peritoneal lavage with RPMI and then plated at the number of 600,000 cells/plate in a 24-well-plate. After 2 h of adhesion, supernatant was removed and macrophages (adherent cells) were recovered. Splenic cells were isolated after spleen digestion at 37°C during 10 minutes with supplemented RPMI with DNAse (10 mg/mL 1%) and D-collagenase (4 mg/mL 25%).

### Flow cytometry cell analysis

Cell suspensions of spleen and peritoneal cells were stained with fluorescent antibodies (Abs) for 10 min at 4°C for flow cytometry analyses. Analysis of 200,000 events gated on viable cells was performed on a BD LSRFortessa Cell Analyzer (BD Biosciences, NJ, USA). Results were analyzed with BD FACSDiva Software 6.0 (BD Biosciences). Fluorescent Abs (TCRβ-Biot, SAV-BV605, CD4-PB, NK1.1-PerCPCy5.5, CD19-FITC, CD45-APC, CD45-BV711, CD11c-PECy7, Ly6C-BV421 and Ly6G-APC) were obtained from BD and CD8-APCvio770, CD11b-FITC and F4-80-PE were obtained from Milteyni.

### Measurement of polymorphonuclear cells (PMNs) oxidative burst

The oxidative burst of PMNs was evaluated through the measurement of reactive oxygen species (ROS) by flow cytometry in peritoneal fluid 16 h after *in vivo* infection by *S.aureus.* Dihydrorhodamine 123 (DHR 123, Sigma Aldrich) was used to detect intracellular ROS. Peritoneal cells were incubated with DHR 123 before incubation with 0.2 μg of PMA or Hanks’ balanced salt solution (HBSS) (vehicle) and then with fluorescent Abs. Erythrocytes were lysed in 1X BD FACS Lysis (BD Biosciences) solution. Samples were then washed and analyzed by BD LSRFortessa Cell Analyzer. Mean fluorescence intensity (MFI) of DHR 123 was measured in PMNs population. Gating strategy is shown in **Figure S1A**.

### Measurement of *in vitro* macrophages phagocytosis by flow cytometry

Mice were treated with MMF or NaCl 0.9% for 4 days, and were euthanized. Peritoneal macrophages were isolated, plated, and after 2 h of adhesion, supernatant was removed and cells were stimulated with Alexa-Fluor 488-*S. aureus* (Molecular Probes, BioParticles^®^), at a multiplicity of infection (MOI) of 10 during 30 minutes. After stimulation, plates were washed and extracellular fluorescent bacteria were quenched with Tryptan Blue 0.4%. After incubation with fluorescent Abs (F4/80), samples were analyzed by BD LSRFortessa Cell Analyzer. Gating strategy is shown in **Figure S1B**.

### Measurement of phagocytosis by confocal microscopy

Mice were treated with MMF or NaCl 0.9% during 4 days and were euthanized one hour after the last injection. Peritoneal macrophages were plated on glass coverslips (BD Biocoat, Bedford, MA) in a 24-well-plate, and after 2 hours of adhesion, supernatant was removed and cells were incubated during 30 minutes with Alexa-Fluor488- *S.*aureus (MOI 10). After being washed in PBS, fixed with 4% paraformaldehyde and washed with 0.1 M glycine, cells were then washed with PBS, 0.2% BSA for 30 minutes and labeled with a specific anti-*Staphylococcus* antibody (Abcam, Cambridge) for 1 hour at room temperature. Cells were subsequently incubated with a secondary antibody and DAPI. All images were obtained using a confocal Leica DMI6000 microscope and analyzed using ImageJ 1.40 software.

### Western Blot analysis

Mice were treated with MMF (20 mg/kg OD) or NaCl 0.9% for 4 days, and were euthanized one hour after the 4^th^ injection. Peritoneal macrophages were then isolated, stimulated or not with 1 x 10^8^ CFU of heat-killed *S. aureus* (HKSA) for 45 minutes and solubilized in a lysis buffer. Protein extracts were solubilized at 95°C in Laemmli buffer and separated by SDS–polyacrylamide gel electrophoresis as previously described (17). Specific antibodies (phospho-p65 (Tyr536), p65 or actin (Cell Signaling, Denver, MA) and secondary antibody were used, and proteins were revealed with a chemoluminescent kit according to the manufacturer’s instructions (SuperSignal™ West Pico Chemiluminescent Substrate, Thermofisher, Waltham, Massachusetts, USA). Densitometry quantification of phospho-p65 was performed with ImageJ^®^ software and was normalized with densities of total p65 for each stimulated condition.

### Statistical analysis

Survival curves were analyzed using the Kaplan-Meier method and compared using the Log-Rank test. Continuous variables were expressed as mean ± SD and compared using the Student t-test and ANOVA with Tukey’s multiple comparisons tests (SPSS software 20.0, IBM, Chicago, IL).

Fluorescent labeled-antibodies and products references are summarized in the **Supplemental Table 1**.

## Results

### Low-dose MMF improves survival during *S. aureus* sepsis

**Figure 1:**
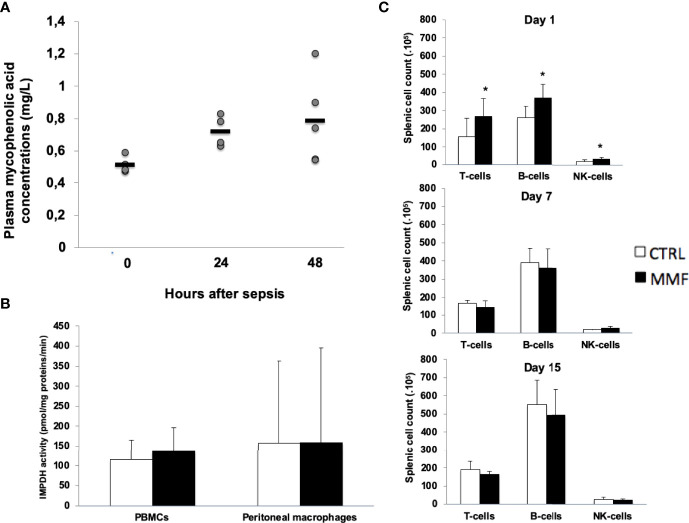
Low-dose MMF did not have any impact on splenic lymphocytes sub-population and IMDH activity during sepsis Mice received MMF i.p. 20 mg/kg once daily, one day before i.p. injection of 10^8^ *S. aureus* and for 4 more days. Trough concentrations were measured at H0 (n=4), H12 (n=4) and H24 (n=5) **(A)**. IMPDH activity was measured in PBMCs and in peritoneal macrophages 4 days after treatment with MMF (or vehicle), one hour after the last injection **(B)**. Splenic lymphocyte sub-populations were counted by flow cytometry, at day 1, 7 and 15 of sepsis **(C)** *P<0,05.

To test the effect of MMF on survival and on innate immune responses in sepsis, we used C57BL/6J mice infected with i.p. 1.10^8^ CFU *S. aureus*, and we developed a protocol of low-dose MMF administration aiming for stable plasmatic MPA concentrations ranging from 0.5 to 1 mg/L, concentrations known to have no effect on lymphocyte proliferation in organ transplant recipients (12). Administration of 20mg/kg once daily intraperitoneally resulted in stable plasma concentrations of MPA in the target range (**Figure 1A**) and did not change IMPDH activity after infection (**Figure 1B**). With this dose, splenic B- and T-cells counts did not decrease after infection (**Figure 1C**).

**Figure 2:**
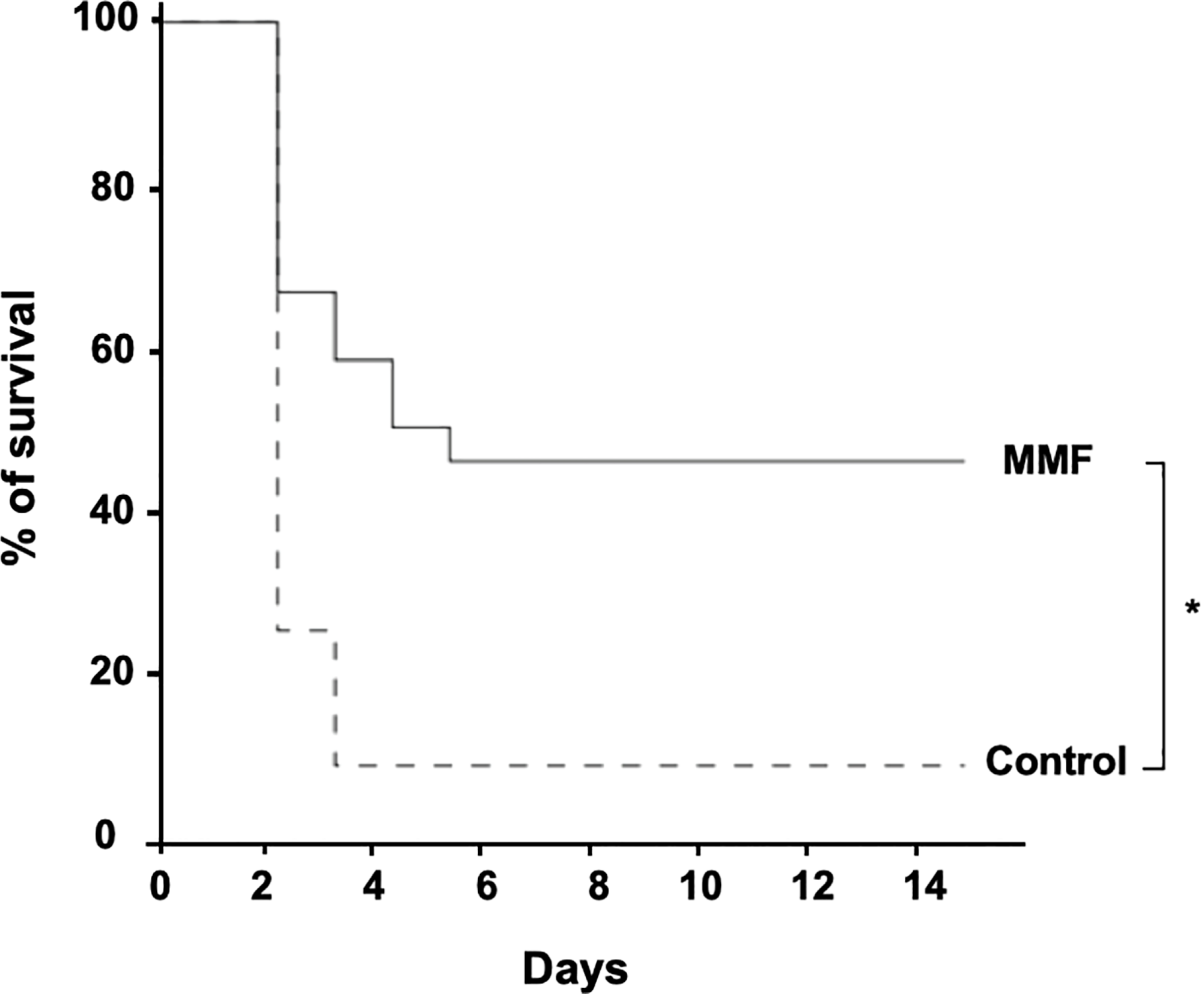
MMF improves survival during i.p. *S.aureus* infection. Mice were treated with MMF, which started one day before i.p. injection of 10^8^ CFU *S. aureus* and for 3 more days (dashed line, n=25) and were compared to control mice treated with an equivalent volume of NaCl 0.9% (Dotted line, n=25). Survival was monitored for 14 days after infection. *Log-rank test, *P*<0.01.

We observed that treatment with MMF significantly decreased sepsis-induced mortality compared to control (52% vs. 92%) (p<0.001) (**Figure 2**).

### Low-dose MMF improves bacterial clearance and attenuates cytokine release during *S. aureus* infection

**Figure 3:**
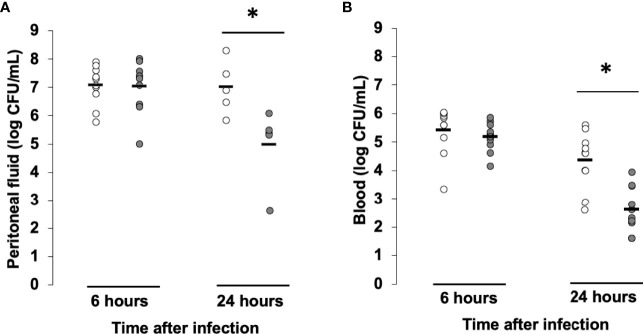
MMF improves bacterial clearance in mice infected with *S. aureus*. Mice were treated with MMF, which started one day before i.p. injection of 10^8^ CFU *S. aureus* (n=10, grey dots) and were compared to control mice treated with an equivalent volume of NaCl 0.9% (control, n=10, white dots). 6 and 24 hours after infection, mice were euthanized and quantitative bacterial culture was assessed in peritoneal fluid **(A)** and blood **(B)**. Differences between MMF and control conditions were calculated using Student’s *t* test. * *P*<0.05.

In order to decipher the mechanisms explaining the protective effect of low-dose MMF in our *S. aureus* induced model of sepsis, we investigated the effect of MMF on bacterial clearance *in vivo*. We determined bacterial counts in the peritoneal fluid and blood of *S. aureus* infected mice in the presence or absence of MMF. As shown in **Figure 3A, B** MMF-treated and untreated mice presented similar CFU numbers in both compartments 6 hours after infection. However, 24 hours after infection, bacterial counts in the peritoneal fluid and in blood were significantly lower for MMF treated mice. We also tested whether MMF had a direct bactericidal effect on *S. aureus*, we incubated *S. aureus* with increasing concentrations of MMF or MPA and measured bacterial load by optical density. We confirmed that MPA had no direct impact on the growth of *S. aureus* (**Figure S2**).

**Figure 4:**
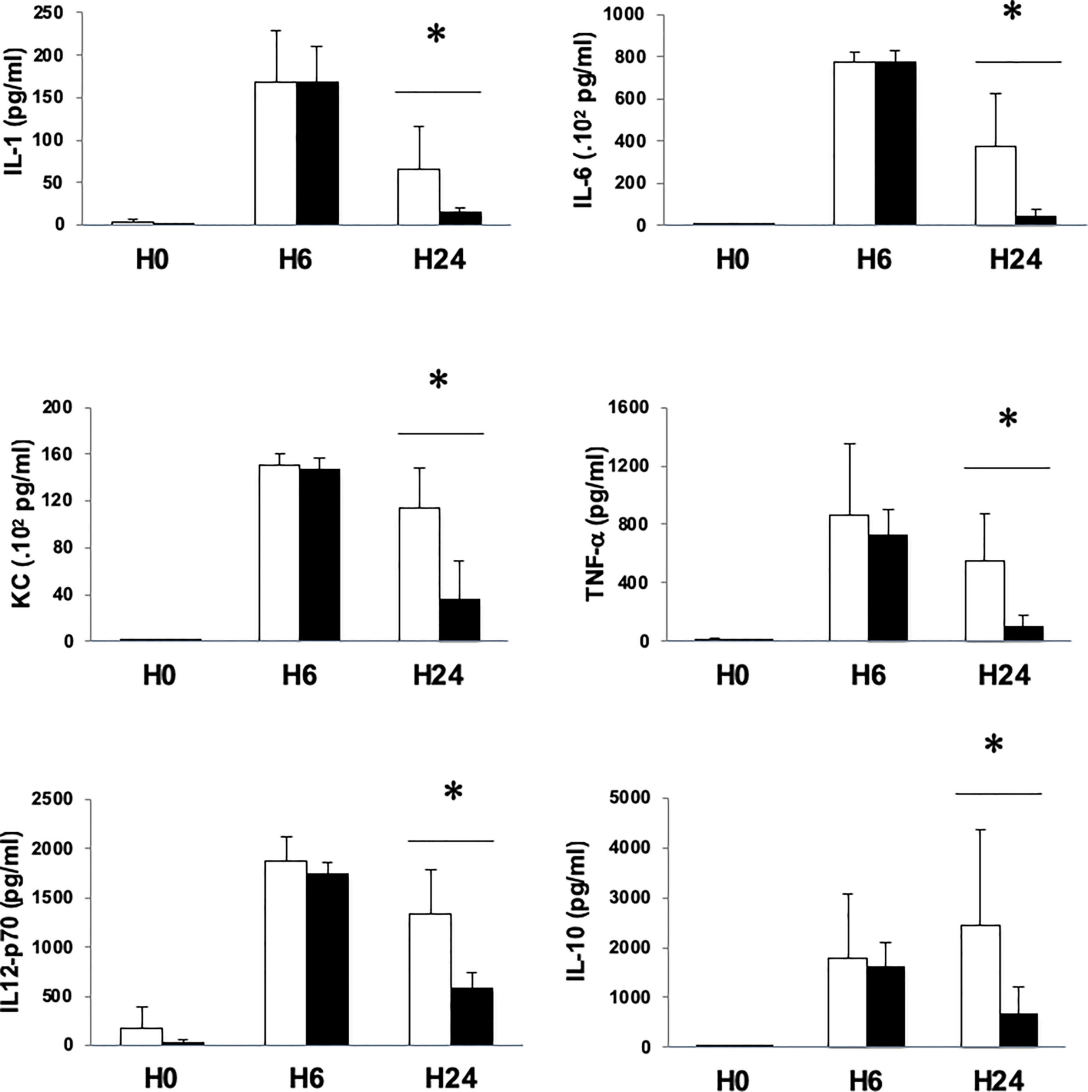
MMF treatment attenuates the release of pro-inflammatory and anti-inflammatory cytokines during infection with *S. aureus*. Mice were treated with MMF, which started one day before i.p. injection of 10^8^ CFU *S. aureus* (black histograms) and were compared to control mice treated with an equivalent volume of NaCl 0.9% (white histograms). Mice were euthanized 6 (n=5 in each group) and 24 hours (n=10 in each group) after infection. Serum IL-1, IL-6, KC, TNF, IL12p70 and IL-10 were measured by Mesoscale Discovery technology. Data are represented as means ± SD. Differences were calculated using Student’s *t* test. * *P*<0.05.

To test whether the decrease of peritoneal and blood bacterial counts observed under MMF treatment had an impact on the inflammatory response, we measured cytokine responses in our model of *S. aureus* sepsis and bacteremia. Whereas cytokine levels 6 hours after infection between MMF-treated mice and control mice were comparable, we observed that 24 hours after infection, all measured cytokines (TNF-α, IL-1β, IL-2, IL-4, IL-5, IL-6, IL12p70, KC and IL-10) were at a significantly lower level in MMF-treated mice, as compared with untreated mice (levels of TNF-α, IL-1β, IL-6, IL12p70, KC and IL-10 are represented in **Figure 4**).

### Low-dose MMF improves phagocytic activity of macrophages during *S. aureus* sepsis

**Figure 5:**
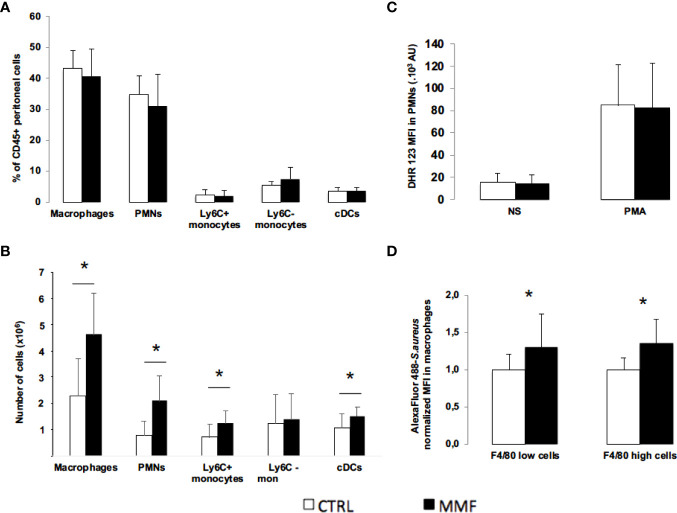
Effect of MMF on innate immune cells distribution and functions during *S. aureus* sepsis Mice were treated with MMF (n=10, black histogram), or with the same volume of NaCl 0.9% (control mice, n=10, white histogram). 24 hours after infection, peritoneal and splenic cells were stained for CD45, CD11b, Ly6C and Ly6G (PMNs), CD11b and Ly6C (monocytes), CD11b and F4/80 (macrophages) and CD11c (conventional dendritic cells) **(A, B)**. Mice were treated with MMF (black histogram, n=14) or the same volume of NaCl 0.9% (white histogram, n=14) and were infected with *S. aureus* one hour after the 2^nd^ injection. Peritoneal cells were collected 16 hours after infection, incubated with DHR 123 and cells were then activated *in vitro* by PMA (0.2 μg). NS represents non-stimulated condition. Peritoneal cells were stained for the PMN markers and DHR 123 fluorescence was measured by flow cytometry **(C)**. Peritoneal cells (6. 10^5^ cells) were isolated from mice treated with MMF (black histogram) or NaCl 0.9% (white histogram) for 4 days, and were incubated 30 minutes with *S. aureus* (MOI 10) stained with Alexa Fluor 488. Cells were then stained with F4/80, and intracellular MFI was measured by flow cytometry in F4/80+ macrophages and F4/80 high macrophages. MFI for each condition was normalized on the average of MFI in control mice for each experiment. Data were representative of 6 experiments **(D)**. Data are represented as means ± SD. Differences were calculated using Student’s *t* test. * *P*<0.05.

To characterize the effect of MMF on cell distribution, we used flow cytometry to count peritoneal and splenic polymorphonuclear (PMN) cells, monocytes, macrophages, and dendritic cells 24 hours post-infection (Gating strategy in **Figure S1**). In absence of *S. aureus* infection, we did not observe any direct effect of MMF on peritoneal and splenic cells proportions (data not shown). 24 hours after *S. aureus* infection, we observed that cell distribution in the intraperitoneal fluid did not differ between MMF-treated and control mice (**Figure 5A**); conversely, MMF-treated mice exhibited a significantly higher number of splenic PMNs, macrophages, pro-inflammatory monocytes and conventional dendritic cells (**Figure 5B**).

As PMNs and macrophages are central to bacterial clearance (18), we studied the impact of MMF on their anti-bacterial functions. We first observed that peritoneal PMNs oxidative activity was similar between MMF-treated and untreated mice (**Figure 5C**). We then assessed *in vitro* phagocytosis capacity of peritoneal macrophages by flow cytometry. Intracellular fluorescence was significantly higher in macrophages from MMF-treated mice as compared to untreated mice (**Figure 5D**). We confirmed this result by confocal microscopy (**Figure S3**). These results suggest that MMF potentiates phagocytic activity of macrophages incubated with *S. aureus.*

### Low-dose MMF effect during *S. aureus* sepsis is dependent on TLR4-NF-κB signaling pathway

**Figure 6:**
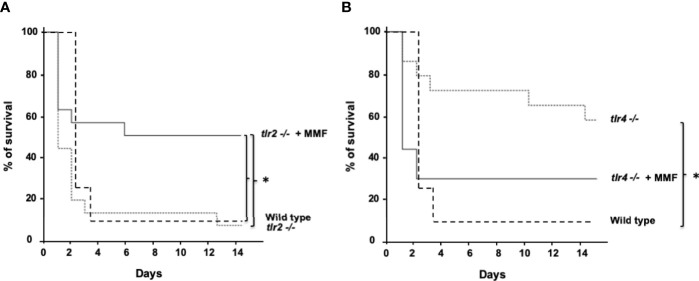
MMF improves survival during i.p. *S. aureus* infection in *Tlr2(-/-)* mice **(A)** but not in *Tlr4(-/-)* mice **(B)** *Tlr2(-/-)* and *Tlr4(-/-)* mice were treated with MMF, which started one day before i.p. injection of 10^8^ CFU *S. aureus* and for 3 more days (dashed line, n=17 and n=15, respectively, representing 4 experiments) and were compared to *Tlr2(-/-)* and *Tlr4(-/-)* control mice (Dotted line, n=17 and n=15 respectively, representing 4 experiments) and with wild type control mice treated with an equivalent volume of NaCl 0.9% (n=25, representing the 8 experiments). Survival was monitored for 14 days after infection. *Log-rank test, *P*<0.01.

Host defense against *S. aureus* infection mostly depends on TLR2-mediated activation of innate immune responses (19), and in a lesser extent on TLR4-mediated activation of innate immune response (20) 18,19). To determine whether the protective effect of MMF on *S. aureus* sepsis depends on TLR2 pathways, we used an i.p. injection of 1.10^8^ CFU *S. aureus* to infect *Tlr2(*-/-) mice treated or not with MMF, and assessed survival. Similar to wild-type animals, we observed that MMF reduced mortality in *Tlr2(*-/-) mice infected with *S. aureus*, suggesting that the protective effect observed with infra-immunosuppressive doses of MMF was independent of TLR2 (**Figure 6A**).

**Figure 7:**
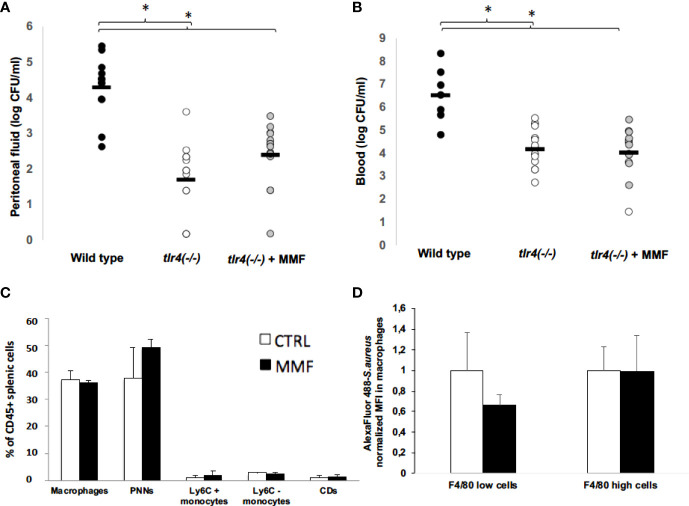
MMF has no impact on bacterial clearance, on splenic and peritoneal cells and on macrophages phagocytosis activity 24 hours after S. aureus infection in *Tlr4(-/-)* mice *Tlr4(-/-)* mice. Mice were treated with MMF, which started one day before i.p. injection of 10^8^ CFU *S. aureus* (n=14, grey dots) and were compared to control mice treated with an equivalent volume of NaCl 0.9% (wild type, n= 12, black dots; *Tlr4(-/-)* control mice, n=14, white dots). At H24 after infection, mice were euthanized and quantitative bacterial culture was assessed in peritoneal fluid **(A)** and blood **(B)**. Mice were treated i.p. with MMF (n=7, black histogram), or with the same volume of NaCl 0.9% (control mice, n=7, white histogram), which started one day before the i.p. injection of *S. aureus*. 24 hours after infection, splenic cells were stained for CD45, CD11b, Ly6C and Ly6G (PMNs), CD11b and Ly6C (monocytes), CD11b and F4/80 (macrophages) and CD11c (conventional dendritic cells) **(C)**. Peritoneal cells (6. 10^6^ cells) were isolated from *Tlr4 (-/-)* mice treated with MMF (black histogram) or NaCl 0.9% (white histogram) for 4 days, and were incubated 30 minutes with *S. aureus* (MOI 10) stained with Alexa Fluor 488. Cells were then stained for the macrophage marker F4/80, and intracellular mean fluorescence of intensity (MFI) was measured by flow cytometry in F4/80+ macrophages and F4/80 high macrophages. MFI for each condition was normalized on the average of MFI in control mice for each experiment. Data were representative of 2 experiments **(D)**. Differences between MMF and control conditions were calculated using ANOVA with Tukey’s multiple comparisons tests and Student’s *t* test. * *P*<0.05.

To determine whether the protective effect of MMF on *S. aureus* sepsis depends on TLR4 pathways, we assessed survival of *Tlr4(*-/-) mice infected intraperitoneally with 1.10^8^ CFU *S. aureus* in the presence or absence of MMF. We found that *Tlr4(*-/-) mice showed lower mortality rates than wild type mice (**Figure 6B**), and peritoneal fluid and blood bacterial loads in *Tlr4(*-/-) mice were significantly lower (**Figure 7A, B**). However, MMF treatment does not improve mortality rate of *Tlr4(*-/-) mice, as compared to non-treated wild-type mice (**Figure 6B**). In *Tlr4(-/-)* mice, bacterial clearance in peritoneal cavity and blood (**Figure 7A, B**), recruitment of innate immune cells in the spleen (**Figure 7C**), and macrophages phagocytosis activity (**Figure 7D**) were not modified by MMF treatment.

*In vitro* models suggested that IMPDHII was a negative regulator, and MPA a potentiator of NF-κB activity under TLR stimulation (17).

**Figure 8:**
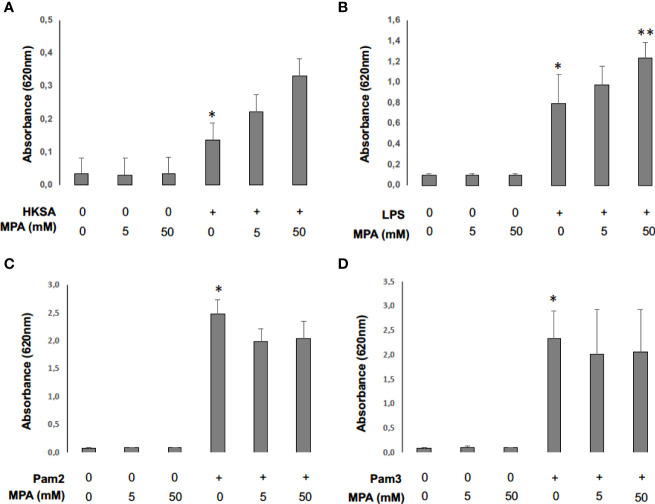
Mycophenolic acid potentiates NF-κB activity following monocyte cell-stimulation by *S. aureus*. THP1-Blue NF-kB cells (2. 10^5^ cells) were incubated 24 hours at 37°C with CO2 5% with different concentrations of MPA and stimulated with HKSA (1 x 10^5^) **(A)**, LPS (0.05ng/μl) **(B)**, Pam2 (0,05ng/μl) **(C)** or Pam3 (0,05ng/μl) **(D)**. Optical density (OD) at 620 nm reflecting NF-κB activity was then read with EPOCH spectrometry in 96-well-plate. Differences between non-stimulated and stimulated conditions and between stimulated conditions with and without MPA were calculated using ANOVA with Tukey’s multiple comparisons tests. * represents *P*<0.05 between non-stimulated and stimulated conditions, ** *P*<0.05 represents *P*<0.05 between stimulated conditions with and without MPA.

To assess whether concentrations of MPA used in our model impact NF-κB activation, we stimulated THP1-Blue NF-κB cells incubated or not with MPA with heat-killed *S. aureus* (HKSA) and different TLR agonists: LPS, Pam2 and Pam3 (21). We observed that HKSA and all tested TLR agonists induced NF-κB activity 24 hours after stimulation. The incubation of THP1-Blue cells with MPA potentialized significantly NF-κB activation by LPS and tended to potentotialize NF-κB activation by HKSA (**Figure 8A, B**). However, MPA did not potentialize NF-kB activation by Pam2 and Pam3 (**Figure 8C, D**).

In order to verify if MMF dose we used was sufficient to potentiate NF-κB activity in our model, we stimulated macrophages of non-infected MMF-treated and untreated mice with HKSA and measured the level of p65 phosphorylation by western blot. We observed that MMF increases phosphorylation of p65 after heat-killed *S.aureus* (HKSA) stimulation (**Figure S4**), suggesting that MMF increases NF-kB activation after HKSA stimulation.

Altogether, these results suggest that the protective effects observed with infra-immunosuppressive doses of MMF depend on TLR-NF-κB signaling pathways and seem to be dependent on the presence of TLR4.

## Discussion

During sepsis, the innate immune system attempts to eliminate the invading microorganism through a potent proinflammatory response. Early deaths may be related to failure to eradicate the pathogen or to an unbalanced and harmful host response that can promote organ dysfunction, highlighting the complexity of immune therapeutic interventions in sepsis. In the present study, we have found that low-dose MMF improved survival in a murine model of *S. aureus*-induced sepsis. Treatment with MMF enhanced bacterial clearance and improved phagocytic capacity of macrophages. These effects may depend on NF-κB signaling, downstream TLR4 in particular. As MMF is known to be an immunosuppressive drug, these results may seem paradoxical. However, several reports indicate that immunosuppression associated with transplantation may provide a survival advantage to transplant recipients with bacteremia and sepsis through modulation of the inflammatory response (22).

Very few studies have assessed the direct impact of immunosuppressive agents such as MMF on sepsis outcomes. Consistent with our results, Assfalg et al. reported in 2010 that MMF used at infra-immunosuppressive doses in combination with antibiotic therapy improve survival as compared to antibiotic therapy alone in a model of polymicrobial sepsis induced by colon ascendant stent peritonitis (11). More recently, Huang et al. reported that MMF used at immunosuppressive doses improved survival and attenuated organ dysfunction in a CLP model of polymicrobial sepsis (10). In this study, treatment with low-dose MMF was associated with increased bacterial clearance, a decrease of inflammatory cytokines and alleviated apoptosis of spleen and peritoneal macrophages. Resident cells are involved in local bacterial killing and alert the host through increased secretion of cytokines/chemokines, which contribute to the recruitment and activation of innate immune cells to the site of infection (2, 18, 23). In our study, MMF treatment of septic mice was associated with a higher rate of splenic PMNs and macrophages, which are very important for pathogen clearance at the early stage of infection and may have contributed to the improved systemic bacterial clearance and subsequent decrease of inflammatory cytokine levels observed in treated mice (18). These findings suggest an impact of MMF on innate immune responses; in contrast, the low doses of MMF used in this study did not affect IMPDHII activity and lymphocytes subpopulations, suggesting a minor impact on adaptive immunity. We also found that MMF enhanced phagocytic activity in macrophages of MMF-treated mice. Activation of PI3-K/AKT signaling is known to be involved in phagocytic activity of macrophages (24) and bacterial killing (25, 26). Toubiana et al. previously showed that under TLR2 stimulation, MPA increased phosphorylation of AKT and subsequent PI3K activation and then NF-κB activity (17). Interestingly, we found that MPA potentialized NF-κB activation by HKSA and a TLR4 agonist (LPS) in THP1 monocytic lineage and that macrophages of MMF treated mice showed an increase of p65 phosphorylation after *in vitro* HKSA stimulation.

TLR2 being the main receptor activated by *S. aureus* (27), we first hypothesized that MMF effect in our model was dependent on TLR2. Interestingly, we observed that the positive effects of MMF on survival and bacterial clearance persisted in *Tlr2*(-/-) mice, suggesting that the impact of MMF in our model does not depend on TLR2. Conversely, we found that *Tlr4*(-/-) mice tended to be protected from death in our model of *S. aureus* sepsis, suggesting that the effect of MMF is at least partially mediated through a TLR4-dependent improvement of the immune response to *S. aureus*, as previously described (20, 28–31). However, the mechanisms involved remain unclear. Ming Chu et al. proposed a model in which *S. aureus* phenol-soluble modulins would act as TLR4 antagonists and inhibit TLR4/NF-κB signaling pathway. Conversely, leucocidin, a *S. aureus* exotoxin was suspected to activate dendritic cells through a TLR4-dependent pathway (30). In our model, mortality of *Tlr4*(-/-) mice treated with low-doses MMF tended to be higher than *Tlr4*(-/-) control mice but this trend did not reach significance. No survival advantage and no improvement in bacterial clearance was observed in treated wild-type mice. In addition, our *in vitro* experiments showed that after stimulation with a specific TLR4 agonist and not after a stimulation with a specific TLR2 agonist, NF-κB activation is potentiated by MPA, suggesting that the effects of MMF effects may be TLR4-dependent. The fact that phagocytosis was not significantly different in *Tlr4^-/-^* mice in the presence/absence of MMF points at alternative mechanisms to explain the survival difference observed in our study. Mechanisms explaining how MMF interacts with TLR4 signaling pathways needs to be deciphered, potentially through the study of MMF biological effects on *Tlr4*(-/-) vs. *Tlr4*(+/+) primary PMN and macrophage cells *in vitro*, or through the use of *Tlr4* knock-out and *Tlr4*-overexpressing monocyte cell lineages. *In vivo*, a comprehensive transcriptomic assessment of the innate immune response could help understand which mechanisms contribute to the phenotype observed.

In conclusion, in our model, MMF is able to improve activation of NF-kB and therefore protects mice infected by *S. aureus* from fatal outcome through enhanced bacterial clearance in local and systemic compartments and phagocyte function. These effects are responsible of a decrease of inflammatory cytokines. The exact cellular and molecular mechanisms underlying MMF effect remain to be elucidated but seem to be dependent on the presence of TLR4.

## Data availability statement

The raw data supporting the conclusions of this article will be made available by the authors, without undue reservation.

## Ethics statement

The animal study was reviewed and approved by Institutional Animal Care and Use Committee and the French Agriculture and Forestry Ministry (APAFiS #12959).

## Author contributions

J-DC and JT supervised the work. J-DC and JT designed the study and the methodology. FA-L, NB, CR, BB and SS realized experiments. FA-L, J-DC and JT analyzed data and drafted the manuscript. All authors reviewed and approved the final version of the manuscript.

## Funding

This work was supported by a research grant from CARISMA. FAL was supported by a grant from Assistance Publique – Hôpitaux de Paris.

## Acknowledgments

The authors thank Abdelouhab Bouaboud and Samuel Bellais for their advice on bacteriological techniques and Matthieu Besnard for taking care of mice.

## Conflict of interest

The authors declare that the research was conducted in the absence of any commercial or financial relationships that could be construed as a potential conflict of interest.

## Publisher’s note

All claims expressed in this article are solely those of the authors and do not necessarily represent those of their affiliated organizations, or those of the publisher, the editors and the reviewers. Any product that may be evaluated in this article, or claim that may be made by its manufacturer, is not guaranteed or endorsed by the publisher.
