# Supplementary material for: Low-dose mycophenolate mofetil improves survival in a murine model of Staphylococcus aureus sepsis by increasing bacterial clearance and phagocyte function
Source: Front Immunol. 2022 Jul 19;13:939213. doi: 10.3389/fimmu.2022.939213 (PMC9351454; doi:10.3389/fimmu.2022.939213)
Supplement: Supplementary file 1 [file Image_1.pdf]

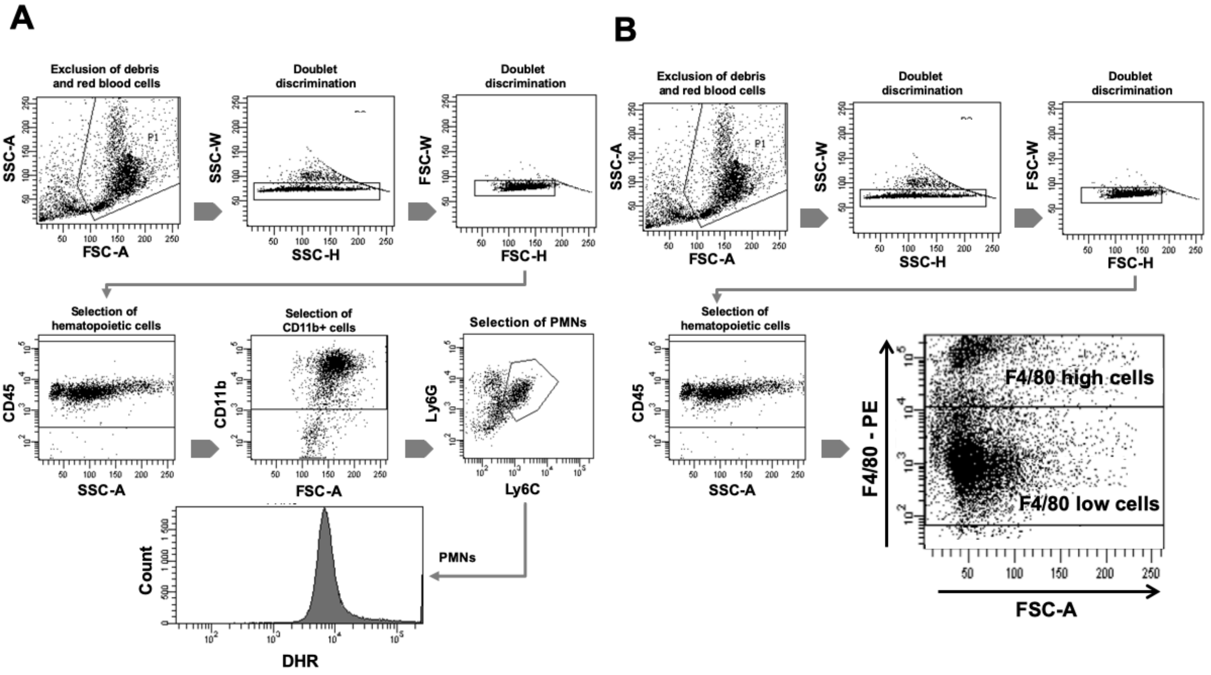

Figure S1. Gating strategies

Gating strategy used to assess oxidative burst of peritoneal polymorphonuclear cells 16 hours after in vivo *S. aureus* infection (**A**). Gating strategy used to assess in vitro phagocytosis activity of peritoneal macrophages with Alexa-Fluor 488 (**B**).

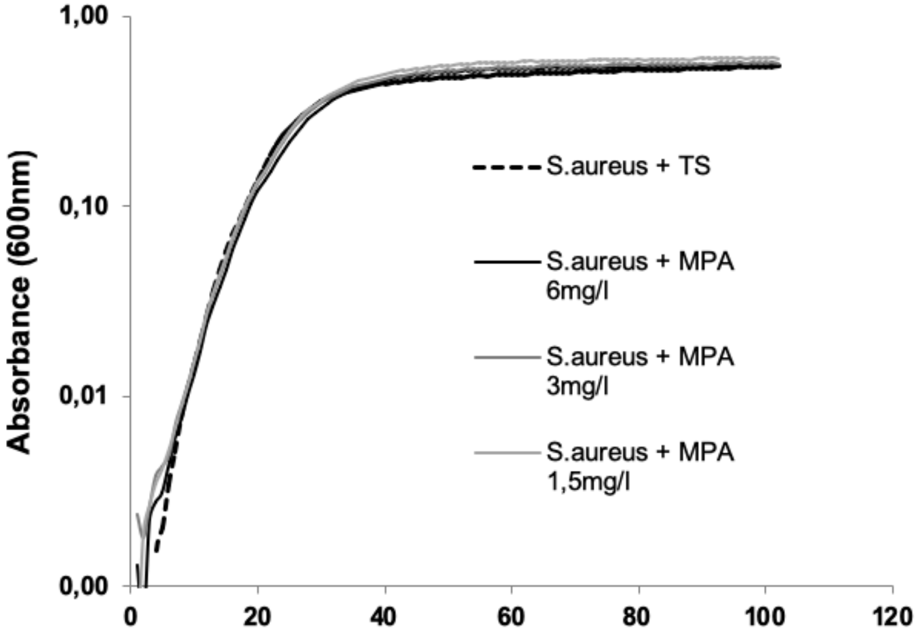

Figure S2. MPA and MMF in vitro effect on *S.aureus* growth

S.aureus were cultured in a tryptic soy broth, and were incubated with a range of concentration of mycophenolic acid and MMF. Absorbance was measured by spectrometer (600 nm) every 10 minutes during 12 hours.

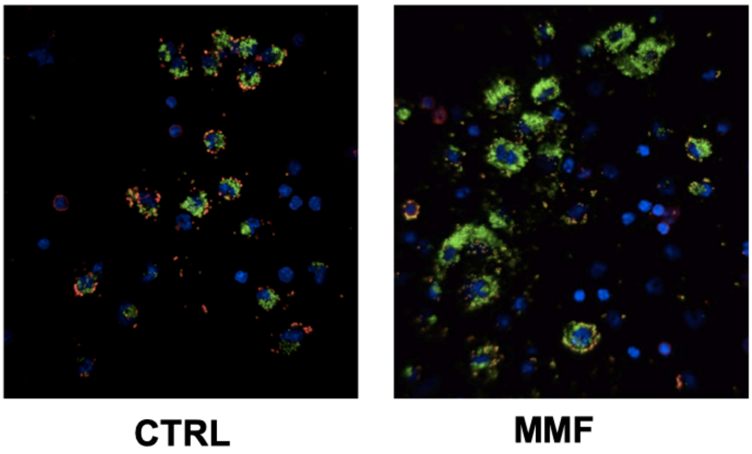

Figure S3. MMF treatment improves in vitro phagocytosis of peritoneal macrophages

Peritoneal cells were plated on glass coverslips and adherent cells were fixed with 4% paraformaldehyde, stained with DAPI, with an anti-staphylococcus antibody and a red fluorescent labeled secondary antibody, and fluorescence was analyzed by confocal microscopy. Data were representative of 3 experiments. Data are represented as means ± SD. Differences were calculated using Student's *t* test. * $P<0.05$.

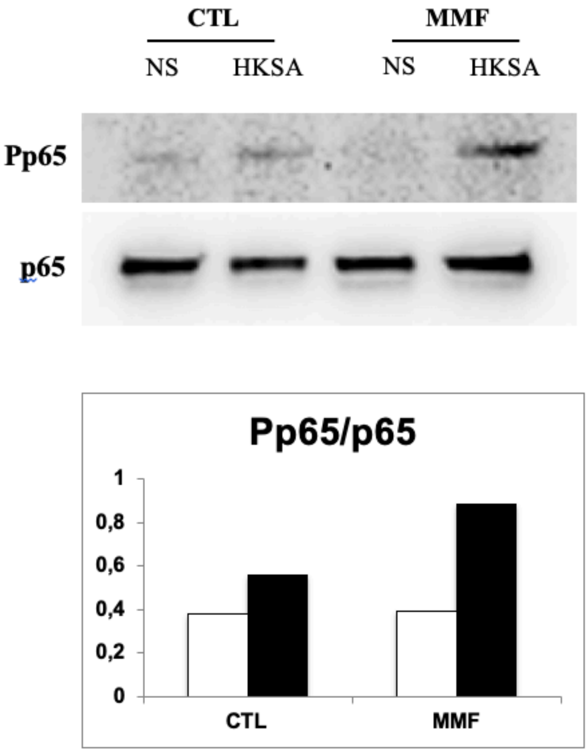

Figure S4. Figure 1. Low-dose MMF potentiates NF-kB activity without decreasing lymphocyte count

Peritoneal cells (1. 10^6 cells) were isolated from mice treated with MMF (black histogram) or NaCl 0.9% (white histogram) for 4 days, and were incubated (S) or not (NS) 45 minutes with 10^8 CFU/ml of heat-killed *S. aureus.* Cells were then lysed and p65 phosphorylation was measured by western blot.
