# Supplementary material for: Low-dose mycophenolate mofetil improves survival in a murine model of Staphylococcus aureus sepsis by increasing bacterial clearance and phagocyte function
Source: Front Immunol. 2022 Jul 19;13:939213. doi: 10.3389/fimmu.2022.939213 (PMC9351454; doi:10.3389/fimmu.2022.939213)
Supplement: Supplementary file 2 [file Table_1.pdf]

Supplemental Table 1. Fluorescent labeled-antibodies and products references

Products	Company	Reference
TCRβ-Biot	BD Biosciences	553169
SAV-BV605	BD Biosciences	563260
CD4-PB	BD Biosciences	558107
NK1.1-PerCPCy5.5	BD Biosciences	551114
CD19-FITC	BD Biosciences	553786
CD45-APC	BD Biosciences	559864
CD45-BV711	BD Biosciences	563709
CD11c-PECy7	BD Biosciences	558079
Ly6C-BV421	BD Biosciences	562727
Ly6G-APC	BD Biosciences	560599
CD8-APCvio770	Miltenyi Biotec	130-102-305
CD11b-FITC	Miltenyi Biotec	130-081-201 130-113-796
F4-80-PE	Miltenyi Biotec	130-102-422 130-116-499
Dihydrorhodamine 123	Sigma Aldrich	D1054-2MG
Mycophenolic acid (powder, BioReagent, suitable for cell culture)	Sigma Aldrich	M3536-50MG
THP1-Blue™ NF-κB cells	InvivoGen	thp-nfkb
V-PLEX Plus Proinflammatory Panel1 Mouse Kit)	Meso Scale Discovery	K15048G-1
Staphylococcus aureus (Wood strain without protein A) BioParticles™, Alexa Fluor™ 488 conjugate	Molecular Probes, BioParticles™ Invitrogen™	S23371
Anti-*Staphylococcus aureus* antibody	Abcam	Ab37644
